# Potassium Channels Blockers from the Venom of *Androctonus mauretanicus mauretanicus*

**DOI:** 10.1155/2012/103608

**Published:** 2012-05-17

**Authors:** Marie-France Martin-Eauclaire, Pierre E. Bougis

**Affiliations:** Aix-Marseille University, CNRS, UMR 7286, CRN2M, Faculté de Médecine secteur Nord, CS80011, Boulevard Pierre Dramard, 13344 Marseille Cedex 15, France

## Abstract

K^+^ channels selectively transport K^+^ ions across cell membranes and play a key role in regulating the physiology of excitable and nonexcitable cells. Their activation allows the cell to repolarize after action potential firing and reduces excitability, whereas channel inhibition increases excitability. In eukaryotes, the pharmacology and pore topology of several structural classes of K^+^ channels have been well characterized in the past two decades. This information has come about through the extensive use of scorpion toxins. We have participated in the isolation and in the characterization of several structurally distinct families of scorpion toxin peptides exhibiting different K^+^ channel blocking functions. In particular, the venom from the Moroccan scorpion *Androctonus mauretanicus mauretanicus* provided several high-affinity blockers selective for diverse K^+^ channels  (SK_Ca_,  K_v_4.x, and  K_v_1.x K^+^ channel families). In this paper, we summarize our work on these toxin/channel interactions.

## 1. The Scorpion Venom Content

Scorpion venoms are very complex mixtures of molecules, constituting a diverse, naturally occurring peptide library, with most peptides displaying different kinds of biological activity [1, 2]. These peptides can specifically bind to a variety of pharmacological targets, in particular ion channels, resulting in neurotoxic effects. Toxins modulating Na^+^, K^+^, Ca^++^, and Cl^−^ currents have been described in scorpion venoms [2].

Toxins that are highly lethal for mammals modify voltage-gated Na^+^ (Na_v_) currents in excitable cells and are referred to as “Na_v_ channel long-chain toxin.” These toxins are single-chain, small, basic peptides (60- to 75-amino-acid residue chain generally folded by four disulfide bridges). They have been described as *α*- or *β*-toxins due to their binding site on Na_v_ channels as well as to their pharmacological effects [1, 3]. *α*-Toxins bind in a voltage-dependent manner on the voltage sensor of the Na_v_ channel domain IV and inhibit the inactivation phase of the action potential. *β*-Toxins act on the channel activation phase by binding to extracellular loops located preferentially on the voltage sensor of the Na_v_ channel domain II (but also occasionally of domain III) [1, 3–6].

Another class of scorpion toxins has also been widely studied, even if these toxins are devoid of serious lethal effect. They block different K^+^ channel subtypes (some of them in the picomolar range) and are so-called “K^+^ channel toxins” [1, 2, 7]. They are usually shorter than Na_v_ channel toxins but are structurally closely related to them. These Na_v_ and K^+^ channels toxins share a common dense scaffold typically formed by an *α*-helix and a *β*-sheet stabilized by disulfide bridges [8].

Several components of scorpion venom that act on Cl^−^ and Ca^++^ channels have also been described [2]. However, they have little or no influence on the venom toxicity for mammals.

This overview will focus, in particular, on our work done on the K^+^ channel blockers purified from the *Androctonus mauretanicus mauretanicus* venom, which were among the first K^+^ channel blockers characterized from scorpion venoms.

## 2. K^+^ Channel Blockers from Scorpion Venoms

K^+^ channels constitute a ubiquitous family of transmembrane proteins which play a key role in the regulation of a wide variety of physiological processes involved in cell excitability, including regulation of heart beat, muscle contraction, neurotransmitter release, hormonal secretion, signal transduction, and cell proliferation [9]. Multiple combinations of K^+^ channels result from the ability of their subunits to coassemble as tetramers, thus considerably increasing the total number of functionally distinct K^+^ channels. According to their functional and gating properties, K^+^ channels have been first divided into four groups: voltage-activated, Ca^2+^-activated, inward rectifier, and two-pore K^+^ channels [10]. Their 3D architecture has now been depicted by X-ray crystallography [11].

K^+^ channel blocker's toxins (KTxs) from scorpion venoms are short peptides, which are made usually of about 28–40-amino-acid residues reticulated by three or four disulfide bridges, forming compact and resistant molecules [7]. They have been invaluable tools for understanding the physiological role of K^+^ channels and have been exploited to gain insights into the structure of the channel pore that they occlude via electrostatic and hydrophobic interactions [12, 13]. They block K^+^ channels from the extracellular side and bind to their outer vestibules. In most cases, they possess at least two functionally crucial residues: examples include a lysine residue that plugs the channel pore with its side chain and a hydrophobic residue that strengthens the interaction between the toxin and its target. These residues are found in very low concentrations in the venoms (from 0.01 to 1% by weight of crude venom) and have almost no toxic effects in mice when injected by subcutaneous route. However, some of them could be very toxic following direct intracerebroventricular injection.

Based on primary amino acid sequences and cysteine pairing, KTxs have been classified into four families, the *α*-, *β*-, *γ*-, and *κ*-KTx [2, 7]. So far more than 120 KTxs, ranging from 23 to 64 amino acids, have been isolated and sequenced. Most of their structures exhibit a common minimal motif, named the “Cystein-Stabilized-Helix” (CSH). As found in Buthidae scorpions, long and short toxins consist of one *α*-helix and two or three *β*-strands, in which two disulfide bridges covalently link a segment of the *α*-helix with one strand of the *β*-sheet structure [8]. Even if their amino acid sequences are different, the conserved peptide fold allows insertions, deletions, and mutations conferring to the toxins diverse selectivity and affinity for their target. Only the recently characterized *κ*-KTxs are formed by two parallel *α*-helices linked by two disulfide bridges [14].

The *α*-KTx family is the largest one and is divided into at least 22 subfamilies, defined according to the primary sequence alignments of the toxins. Each member has diverse, specific blocking activities against voltage-gated (K_v_) and calcium-activated (K_Ca_) channels. Most of the *α*-KTxs bind to the ion channel vestibule through the *β*-sheet side of their structure.

Other, longer peptides, with 45- to 68-amino acid residues and cross-linked by three disulfide bridges, have been characterized and classified as the *β*-KTx family. They possess two structural and functional domains: an N-terminal *α*-helix (with cytolytic or antimicrobial activity like the insect defensins) and a tightly folded C-terminal region with the CSH motif (displaying K^+^ channel-blocking activities [15]). Finally, the *γ*-KTx family was described as specifically targeting hERG channels [16].

## 3. The *Androctonus mauretanicus mauretanicus* Venom

Scorpion stings in Morocco are the primary cause of envenomation and constitute a largely underestimated health problem. An epidemiologic study of four regions of the Moroccan Kingdom, where scorpion stings are prevalent, showed that the stings are mainly due to the black scorpion *Androctonus mauretanicus mauretanicus* (83% of the reported cases). Children, in desert areas far from medical centers, were the primary victims, with casualty rates up to 8% in those under ten years old. The *Androctonus mauretanicus mauretanicus* venom is one of the most toxic Buthidae venoms ever described (its median lethal dose ranges from 0.05 to 0.2 mg/kg by subcutaneous injection in mice) and immunotherapy remains the treatment of choice [17].

Previous fractionation studies of the venom allowed identification of several toxins that are active on different Na_v_ or K_v_ channels [18–22]. At least several major proteins, considered highly toxic to mice, have already been purified and chemically and pharmacologically characterized as classical *α*-toxins [18, 19]. All together, these toxins represent about 28% of the absorbance (wavelength 280 nm) of the crude venom and 73% of the total lethality for mice. The most represented and the most lethal classical *α*-toxin Amm V alone constitutes 11% of the absorbance and 47% of the total lethality.

However, other toxins isolated from the *Androctonus mauretanicus mauretanicus* venom gained popularity as powerful tools because they have displayed some of the highest binding affinity and specificity for K^+^ channels. They have been extensively used to investigate the mechanisms of ion conduction and channel selectivity, as well as the architecture of the pore region. Finally, significant advances have been made by using solid-state NMR data to construct 3D structures from Kaliotoxin (KTX) in complex with a chimeric K^+^ channel KcsA-K_v_1.3 [13]. These studies allowed direct investigation of the molecular rearrangements associated with KTX binding on both the channel selectivity filter and the KTX itself.

**Figure 1:**
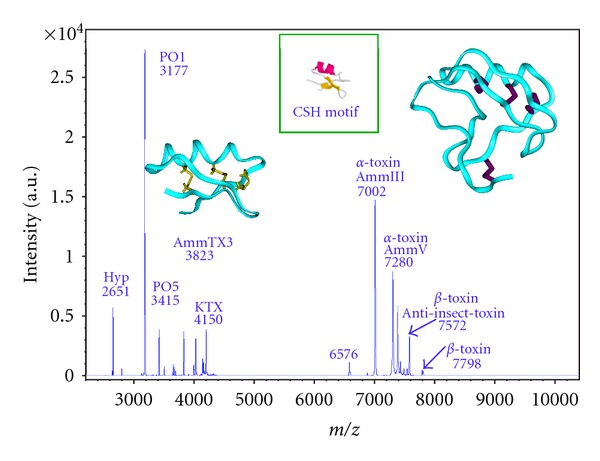
MALDI-TOF mass spectra of pool venom manually extracted from *Androctonus mauretanicus mauretanicus*. K^+^ channel blockers and voltage-gated Na^+^ channels modulators are indicated. P01 and P05, blockers of SK_Ca_ channels; AmmTX3, blockers of K_v_4 and hERG channels; KTX, K_v_1.1, and K_v_1.3 blockers. Amm III and Amm V are major lethal *α*-toxins; *β*-toxins specific for insects are also mentioned. The 3D structure of KTX (left) and of the *α*-toxin of reference AaH II from *Androctonus australis* (right) are shown [13, 24, 25]. The Cys-Stabilized-Helix (CSH) motif is shown in inset [8].

A recent MALDI-TOF mass spectrometry (MS) study has provided new information about the molecular composition of scorpion venom [23]. Through the developments in proteomics, MS is now widely used for accurate and sensitive determinations of molecular masses and identification of heterogenous complex mixtures such as crude venoms. Using an offline MALDI-TOF/MS analysis, we were able to determine the molecular masses of about 70 to 80 different compounds ranging between 3000 and 8000 Da in an *Androctonus mauretanicus mauretanicus* venom obtained under manual stimulation from a pool of animals kept alive at the “Pasteur Institute” of Casablanca, Morocco.  Figure 1 presents the snapshot of this crude venom.

## 4. The Smallest Toxins Identified so Far in the **Androctonus mauretanicus mauretanicus ** Venom: P01 (***α***-KTx8) and P05 (***α***-KTx5), Ligands of SK_Ca_ Channels

**Figure 2:**
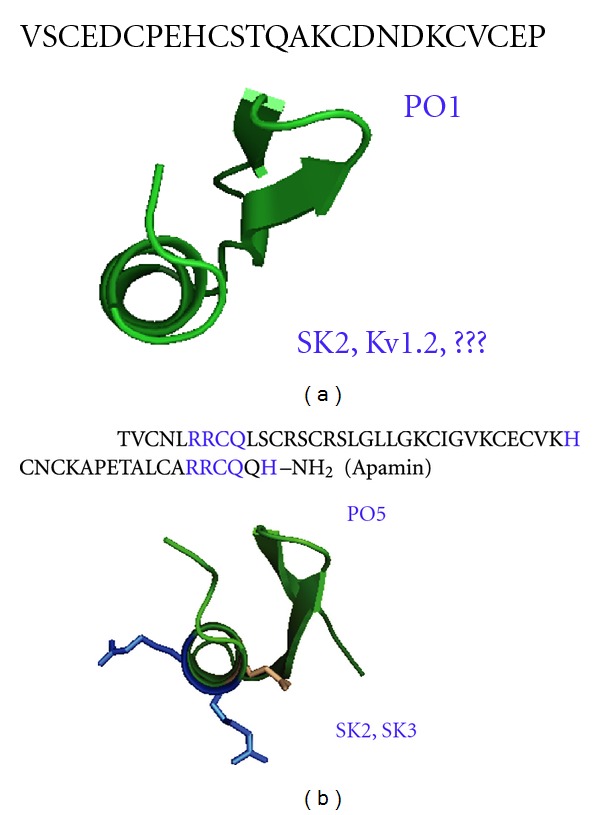
Amino acid sequences and 3D structures of P01 and P05. (a) Amino acid sequence (single letter notation) and 3D structure of P01 [26, 28]. (b) Amino acid sequence (single letter notation) of P05 compared to that of the bee venom apamin (18 mers peptide). Amino acids common to P05 and apamin and crucial for the SK_Ca_ blockade are in blue. These amino acids are materialized on the 3D structure of P05, view by the *α*-helix axe. The two positive Arg are in blue and the neutral Gln is in brown.

P01 (*α*-KTx 8.1 subfamily, 3177 Da) is devoid of significant toxicity in mouse [26]. This toxin of 28-amino-acid residues, which is the smallest K^+^ channels ligand in the *Androctonus mauretanicus mauretanicus* venom, is slightly negatively charged with acidic amino acids localized at the beginning of the *β*-turn and extending along the *α*-helix (Figure 2(a)). Surprisingly, P01 presents exactly the same sequence in *Androctonus mauretanicus mauretanicus, Androctonus australis*, *Androctonus amoreuxi*, and *Buthus occitanus* venoms. Usually, the polymorphism among scorpion toxins is so high that their sequences vary from a scorpion subspecies to another, even in the same structural, pharmacological, and immunological family. P01 is a poor-affinity ligand (300 nM) for the apamin-binding site on rat brain synaptosomes. In comparison, apamin purified from the bee venom displays a very high affinity (K_d_ = 8 pM) for its target, that is, the small-conductance calcium-activated K^+^ channel (SK_Ca_) channel so-called apamin-sensitive channel. Since the first P01 characterization, other closely related analogs have been purified from other Buthidae venoms. Among them, one is particularly interesting because it shares 89% of identity with P01. This analog, called OdK1, was purified from the Iranian scorpion *Odontobuthus doriae* (*α*-KTx 8.5 subfamily). OdK1 is able to block K_v_1.2 channels expressed in oocytes with a median inhibition concentration (IC_50_) value of 183 nM and has no effect on K_v_1.1, K_v_1.3, K_v_1.4, K_v_1.5, and the *Shaker* channels [27]. It is important to mention that P01 has not been tested yet on K_v_1.2 channels in electrophysiological experiments. P01 was only shown unable to compete with ^125^I-KTX (a K_v_1.1 and K_v_1.3 and not K_v_1.2 blocker) bound to its receptor on rat brain synaptosomes. Thus, we cannot totally exclude that P01 could exhibit a poor K_v_1.2 blocking activity. In the same line, OdK1 activity on SK_Ca_ channels has not been tested yet either.

In contrast to P01, P05, another toxin from *Androctonus mauretanicus mauretanicus,* is a high-affinity selective ligand (K_d_ = 100 pM) for the apamin-binding site on rat brain synaptosomes, and it has no effects on BK_Ca_ or K_v_ channels. P05 is a 31-amino-acid long peptide (3415 Da), which belongs to the *α*-KTx5.x's family (as Leiurotoxin1 or Scyllatoxin from the venom of the scorpion *Leiurus quinquestriatus Hebraeus*) (Figure 2(b)) [29, 30]. The toxins from this family have only two-stranded *β*-sheet because of their short N-terminal side [28]. P05 is highly toxic (14 ng, i.e., 4 pmols for a 20 g mouse) and leads to an epileptic behavior when injected in mouse by the intracerebroventricular route. P05 possesses a short stretch of four amino acids similar to the one in the apamin sequence, which constitutes a highly positively charged region in the *α*-helix (containing in particular two Arg residues, Arg_7_ and Arg_9_) exposed to solvent. Extensive structure-function studies using chemically synthesized analogs revealed that these two Arg were critical for P05's interaction with its target as described for apamin [31]. When Arg were replaced by Lys, the activity dropped by a factor of one hundred, and when they were replaced by Ile, the affinity decreased even more to a Kd value in the micromolar range. Moreover, *α*-amidation of His in P05 C-terminal conferred a large gain of function leading to an almost irreversible binding to its receptor [21]. When the apamin C-terminal residue is in the free carboxylic form, only 0.06% of the pharmacological activity of the native C-amidated apamin is retained [32]. Both Arg and C-terminal His create a strong positive electrostatic potential, which drives the toxin to negative residues (Asp) of the channel pore. Using homology-modeling models of SK channels (rsk1, rsk2, and rsk3) and Brownian dynamics methods, the recognition between P05 and its targets was investigated [33]. It was found that the rsk2 channel, presenting the highest frequencies and lowest electrostatic interaction energies, was the most favorable target for P05 binding, while rsk3 was intermediate, and rsk1 was the least favorable. From the P05-rsk2 complex model, it was shown that P05 probably locates around the extracellular pore of SK channels and assures the contact with rsk2 channel using critical basic amino acid residues in its *α*-helix: Arg_6_ (P05)-Asp_364_ (SK), Arg_7_ (P05)-Asn_368_ (SK), and Arg_13_ (P05)-Asp_341_ and Asp_364_ (SK). Further refinements of P05-rsk2 complex model using molecular mechanics showed that six hydrogen bonding interactions exist between P05 and the rsk2 channel. These simulation results were in good agreement with our previous *in vivo* binding experiments and could explain the interaction between P05 and SK channels at the level of the molecular structure.

## 5. The Most Noted K^+^ Channel Blocker Characterized in the **Androctonus mauretanicus mauretanicus ** Venom: Kaliotoxin (***α***-KTx3 Subfamily)

The Kaliotoxin (KTX, 4150 Da) purified from the venom of the *Androctonus mauretanicus mauretanicus* was the first identified member of the *α*-KTx3.1 subfamily, which is currently composed of 13 highly homolog members sharing more than 75% sequence identity. KTX was first described as a BK_Ca_ low-affinity blocker [20], but further analysis showed that KTX blocked more specifically K_v_1.3 channels with a very high affinity (K_d_ = 10 pM) and K_v_1.1 with a much weaker affinity (in the 10 nM range). KTX also exhibited a functional blockade of Ca^2+^-activated K^+^ Gardos channel from rabbit or human red blood cells with a median inhibition concentration value of 5 nM. Interaction between this channel and other peptide toxins indicated that the Gárdos channel was pharmacologically different in several interesting ways from the other Ca^2+^-activated K^+^ channels [34]. Interestingly, KTX was not toxic in mice by subcutaneous injection up to 500 micrograms (100 nmols), but intracerebroventricularly injected mice showed tremor, paralysis, and death with a median lethal dose of only 24 ng of KTX (about 6 pmol for a 20 g mouse).

KTX was then widely used by different international groups to probe the vestibule topology from the lymphocyte K_v_1.3 channel. The homotetrameric K_v_1.3 channel controls the resting potential membrane in T cells and plays a crucial role in human T-lymphocyte activation [35]. Its inhibition causes depolarization and an attenuation of the rise in the intracellular Ca^2+^ concentration that is required for T-cell activation. Accordingly, K_v_1.3 channel is a good therapeutic target for the development of immunosuppressant drugs. It is important to note that K_v_1.3 channel is a key mediator of multiple sclerosis, type 1 diabetes mellitus, and rheumatoid arthritis pathologies [36]. In experimental autoimmune encephalomyelitis (EAE) rat, animal model for multiple sclerosis, KTX was capable of immunosuppressant activity *in vivo*. Addition of KTX during Antigen/T-cell activation led to a large reduction of the T-cell proliferative response, a decreased encephalitogenicity of T cells, and finally improved the symptoms of EAE [37].

A new therapeutic approach for inflammatory bone resorption by targeting K_v_1.3 was also investigated and the potential effect of KTX was tested on inflammatory lesions of periodontal disease. These lesions contain abundant activated/memory T and B cells, which control immunological interactive networks and accelerate bone resorption. Systemic KTX administration finally resulted in an 84% decrease of the bone resorption. These results suggested that KTX could also constitute a potential therapy to prevent alveolar bone loss in periodontal disease [38].

Monoiodinated derivative of KTX (^125^I-KTX) binds specifically to rat brain total membranes with a maximal binding capacity of about 14 fmol/mg of protein and with a high affinity (K_d_ = 80 pM). The distribution of ^125^I-KTX binding sites in rat brain was first studied using quantitative autoradiography on adult brain tissue sections. A comparison with the distribution of K_v_1.1 and K_v_1.3 *α*-subunits by immunohistochemistry or *in situ* hybridization suggested that KTX recognizes channels containing these subunits [39]. Further, we used KTX injected by intracerebroventricular route to rat brain in order to investigate the involvement of K_v_ channels containing K_v_1.1 and K_v_1.3 *α*-subunits in olfactory associative learning and memory. KTX facilitated cognitive processes as learning, in particular in a reference representation [40]. Therefore, it is likely that KTX-sensitive K_v_ channels contribute to the repolarization of action potentials at presynaptic terminals of hippocampal inhibitory neurons and induce facilitation of the transmission.

**Figure 3:**
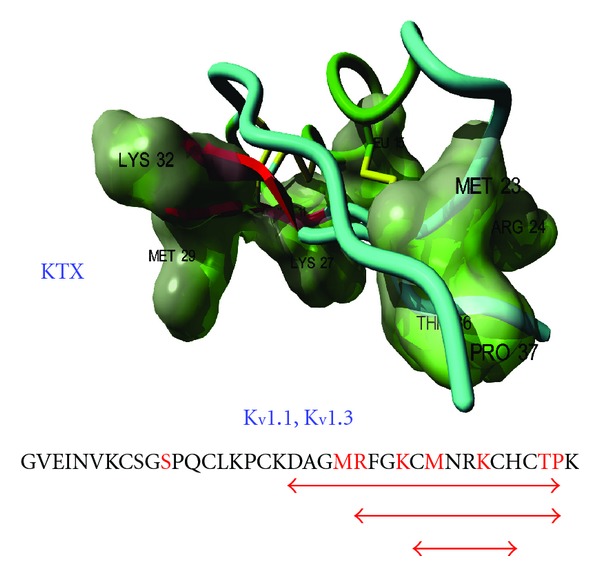
Amino acid sequence and 3D structure of KTX. The crucial amino acid residues involved in the receptor recognition are in red and are materialized on the 3D structure according to Lange et al., 2006 [13]. The *α*-helix is in green, the *β*-sheet in red. Arrows in red indicate the three peptides able to compete (K_d_ = 100 nM) with the ^125^I-KTX bound to its binding site on rat brain synaptosomes [41].

Concerning the KTX structure-function relationship studies, they were first performed using synthetic analogs such as KTX_(1–37)_, KTX_(1–37) )-amide_ and short peptides including KTX_(27–37)_, KTX_(25–32),_ and KTX_(1–11)_. Concerning the short peptides, which corresponded to secondary structural elements, only KTX_(27–37)_ and KTX_(25–32) _were able to compete with ^125^I-KTX for its receptor on rat brain synaptosomes and act as antagonists of KTX. These results demonstrated for the first time that the C-terminal region, particularly the toxin *β*-sheet, was involved in the interaction with the receptor and the channel blockade [41].  Figure 3 summarizes these results.

Several international groups also produced KTX and numerous mutants using chemical synthesis or heterologous expression, in order to identify KTX residues implicated in the specific interaction with K_v_1.3 channels. It was found that the side chain of Lys_27_ of the toxin enters deeply into the channel pore and interacts with the Asp_402_ residue of each subunit [24]. Briefly, the KTX residues involved in the interaction with the channel were Arg_24_, Phe_25_, Lys_27_, Met_39_, Asn_30_, Arg_31_. All these residues were present in the short peptides that we first used to demonstrate the *β*-sheet importance in the channel blockade. A model also predicted that Ser_11_ of the KTX made steric contacts with His_404_ and Pro_405_ of the *α*-subunit IV of the K_v_1.3 and Thr_36_ with His_404_ of the opposing K_v_1.3 *α*-subunit [42].

KTX binding to a chimeric K^+^ channel (KcsA-K_v_1.3) in proteoliposomes was investigated using solid-state nuclear magnetic resonance (ssNMR) [13]. Upon complex formation, significant chemical shift changes of the residues implicated in the specific interaction were observed for both KTX and chimeric channel. For KTX, the conformational changes involved mainly *β*-sheet contacts between the first and third *β*-strand and for the chimeric channel it affected the conformation of both the pore helix and the selectivity filter. The backbone conformation of Kcsa-K_v_1.3 selectivity filter adopted a novel structure with features of both the conducting and collapsed conformation of the KcsA. The ssNMR data directly showed that Asp_64_ in the KcsA-K_v_1.3 vestibule represented an important interaction site for KTX. Large chemical shift changes were seen for Gly_77_, Tyr_78,_ and Gly_79_ in the selectivity filter and also for the side chains of Glu_71_ and Asp_80_ that form carboxyl-carboxylate pairs on the backside of the filter [13]. Finally, an enhanced backbone mobility was detected for two glycine residues within the selectivity filter that are highly conserved amongst potassium channels and are of core relevance to the filter structure and ion selectivity [43, 44].

Combination of additional ssNMR studies, dynamic simulations, and electrophysiological measurements finally revealed the complete mechanism of KTX binding to its receptor site and showed a structural link between inactivation and block of the channel. A mechanism of cooperative toxin-induced conformational changes that are structurally and functionally related to recovery from C-type inactivation, would be the consequence of the very tight interaction between KTX and Kcsa-K_v_1.3. The KTX affinity was lowered by about 20-fold when the K_v_1.3 channels entered in C-type inactivation probably due to changes of the interaction surface between the toxin and the channel [43–45].

All these recent ssNMR data obtained between the chimeric KcsA-K_v_1.3 channel and KTX have largely contributed to decrypt the intimate interaction between a K^+^ channel blocker and its target, as well as to better understand the blockade of the K^+^ conduction.

## 6. The ***α***-KTx_15_ Subfamily: Janus K_v_4.x and hERG Blockers

**Figure 4:**
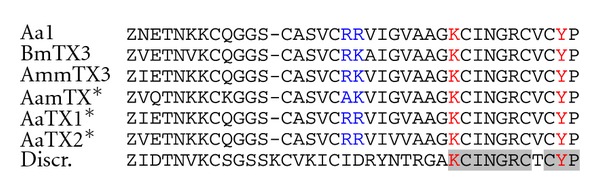
Amino acid sequences of the toxins from the *α*-KTx_15_ subfamily. Aa1 [46], AaTX1, and AaTX2 [48] are from *Androctonus australis*; BmTX3 [47] is from *Buthus martensii*; AmmTX3 [22] is from *Androctonus mauretanicus*; AamTX [49] is from *Androctonus amoreuxi*. Discrepin [54] is from the Venezuelan scorpion *Tityus discrepans*. Single letter notation is used for amino acid sequences. Z is pyroglutamic acid. *Indicates putative amino acid sequence deduced from cloned cDNA oligonucleotide sequence. The *β*-sheet consensus sequence is in grey.

The first member of the *α*-KTx_15_ subfamily characterized was Aa1 from the *Androctonus australis* venom (3869 Da) [46]. At the primary sequence level, the toxin has an unusual N-terminal pyroglutamic acid, like Charybdotoxin and Iberiotoxin, but the rest of its sequence was totally original. Aa1 completely blocked a fast *I*_A_-type K^+^ current from cerebellum granular cells. Using whole-cell patch clamp recording on striatal neurons in culture, we selected another novel toxin, BmTX3 from *Buthus martensii*, which was also able to block a fast *I*_A_-type K^+^ current. The sustained current was unaffected with a micromolar dose of toxin whereas the *I*_A_-type K^+^ current completely disappeared independently of the membrane potential. Autoradiograms of adult rat brain sections demonstrated a highly heterogeneous distribution of ^125^I-BmTX3 binding sites throughout the adult rat brain. High density of receptors was found in the striatum, the CA1 and CA3 field of the hippocampus, the superior colliculus, and in the granular layer of the cerebellum [47]. We then purified AmmTX3 (3827 Da), an analog of Aa1 and BmTX3, from the venom of *Androctonus mauretanicus mauretanicus* [22]. Latter, several cDNAs encoding two Aa1 isoforms, AaTX1 (3867 Da) and AaTX2 (3853 Da), were identified by PCR amplification from a venom gland cDNA library of *Androctonus australis* [48]. Also, another oligonucleotide sequence, AamTX (3751 Da), was amplified from a venom gland cDNA library of *Androctonus amoreuxi* [49]. Altogether these toxins constitute the first members of the *α*-KTx_15_ subfamily (Figure 4). From a pharmacological point of view, theses toxins were unable to compete with any other already described toxins purified from animal venom. However, they were toxic in mouse at high doses by intracerebral injections and induced epileptic status, which could last 24 to 48 hours. Also, they all shared the same target on rat brain synaptosomes because they competed with each other for the same binding site. The nature of the K^+^ channels blocked by AmmTX3 was assessed by performing whole-cell patch recording of the K^+^ currents of striatal neurons and of cerebellum granular cells in culture. In all cases, AmmTX3 inactivated the transient A-current without affecting the sustained K^+^ current, as observed for Aa1 and BmTX3. As well described, A-type K^+^-currents result mainly from the expression of voltage-dependent K_v_ *α*-subunits (K_v_1.4, 3.4, 4.1, 4.2, 4.3) or from the association of K_v_ *β*-subunit with K_v_1 *α*-subunits [50, 51]. In the cerebellum granular cells, the voltage-gated K^+^ channels K_v_4 of the Shal subfamily elicits A-type currents. These channels are fast transient K^+^ channels that regulate the kinetics of the action potential [51, 52]. The localizations of K_v_4 subunits and their auxiliary subunits were similar to the distribution of BmTX3 binding sites in numerous regions of the brain. For example, the high density of BmTX3 binding sites in the CA1 field and CA3 field followed K_v_4.2 and K_v_4.3 immunostaining patterns. In the molecular layer of the dendate gyrus, the BmTX3 binding pattern was also similar to the immunoreactivity pattern of K_v_4 and KChIP subunits, with a higher density in distal dendrites of the granular layer than in the more proximal dendrite areas [53].

Finally, electrophysiological analysis of mammalian cells expressing different A-type channels showed that BmTX3 completely inhibited the rapidly activating and inactivating K_v_4.1 current in a voltage-independent manner. This inhibition was less effective on K_v_4.2 and K_v_4.3 channels and the toxin did not show any effects on other transient currents elicited by K_v_1.4 and K_v_3.4 [53]. Recent electrophysiological studies using acute coronal midbrain slices and AmmTX3 confirmed that the targets of the *α*-KTx_15_ subfamily are K_v_4 channels [55, Rudy B, personal communication]. Interestingly, the radioiodinated *α*-KTx_15_ toxins bound to their receptor on rat brain neurons were not displaced by any spider toxin, which block K_v_4 channels. This result suggests that these scorpion and spider K_v_4 blockers have different binding sites. The *α*-KTx_15_ toxins most probably block the K_v_4 channel pore because tarantula toxins inhibit K_v_4 channels by binding to the voltage-sensor paddles (crucial helix-turn-helix motifs within the voltage-sensing domains composed of the S3b and S4 helices) [56]. Moreover, deletions of the last two BmTx3 C-terminal residues (*α*-KTx_15_ subfamily) were made to assess the role of the penultimate Tyr residue in the receptor recognition. This Tyr resembled to the canonical dyad previously proposed as necessary to block the K^+^ channel conduction with high efficacy. Our results showed that the truncated toxin bound to its receptor less efficiently than the wild-type toxin (by a factor of about 10^5^) and had no more channel blocking activity [57]. All these results suggest that the K_v_4 channels blocked by the *α*-KTx_15_ subfamily might have a canonical K^+^ channel pore structure.

Moreover, we also described a significant hERG-blocking activity from the *α*-KTx_15_ toxins, as previously shown for the *γ*-KTx peptides. AmmTx3 induced a hERG channel block with no alteration of the gating kinetics. According to a model of the *γ*-KTx toxin BeKm-1 from *Buthus eupeus* docked on hERG channels, the toxin BeKm-1 is above the pore entrance and none of its side chains penetrate deeply into the pore. While *α*-KTx members usually interact with channels through their *β*-sheets, *γ*-KTxs modulate hERG in a different way than the one proposed for the interaction between Charybdotoxin and the Shaker channel or between KTX and the K_v_1.3, in which the critical Lys_27_ protrudes into the pore of the channel. BeKm-1 uses its *α*-helix and the following turn (possessing two basic residues Lys_18_ and Arg_20_), to interact with hERG channel [58, 59]. In contrast to the other *α*-KTx_15_ members, a new toxin of this subfamily isolated from the Venezuelan scorpion *Tityus discrepans,* called Discrepin (*α*-KTx_15–6_), was also able to block an A-type K^+^ current in cerebellum granular cells in culture, but was ineffective to block the hERG channel. Its amino acid sequence displays only 50% identity with the other members purified mainly from Old-World scorpion venoms (Figure 4). Discrepin C-terminal *β*-sheet, supposed to interfer with the K_v_4 channel pore, presented a consensus amino acid sequence similar to those found in the other members (in grey on Figure 4), but its “hot spot” for hERG channel blockade was altered. After several point mutations in AmmTX3 and introduction of positive charged residues in Discrepin, it was finally demonstrated that a common “hot spot” composed of two basic residues (Arg_18_ and Lys_19_ near the end of the *α*-helix) conferred hERG blockade activity of *α*-KTx_15_ peptides [54]. From a structural point of view, we proposed that two separate functional surfaces (A and B) could coexist on the *α*-KTx_15_ toxins, and were responsible for two different K^+^-current-blocking functions [60].

To extend our theory to other members from *α*-KTx subfamily, the effects of a “hot spot-” bearing toxin were tested on hERG channels and compared to the results obtained with a toxin without the “hot spot.” From these studies, it was concluded that only the *α*-KTxs possessing the “hot spot” were able to interact with the pore of hERG channels. This pharmacophore could either be -CKKX- or -CKXK- or -CXKKX-, with C being the third Cys, X being any nonpositively charged amino acid, and K being Lys or Arg [54].

## 7. Conclusion

Scorpion venom still remains a proven resource for the discovery of novel biologically active compounds, especially for the pharmacologists involved in research on ionic channels. A total of about 210 *α*-KTx oligonucleotide or amino acid sequences are now referenced in the UniProtKB data bank, but only some of these *α*-KTx peptides have really been shown able to block K^+^ currents. Unfortunately, very often, there is no direct evidence described for the function of the reported peptides. Some of them represent new analogs of well-known families with described channel selectivity, but others exhibit novel structural features or activities. Majority of the *α*-KTx effects were determined on the K_v_1.x subfamily or in a less extent on the Ca^2+^-activated K^+^ channels (SK_Ca_ sensitive to the bee venom apamin or BK_Ca_). During the last two decades, we obtained a lot of results that provided new insight into the targets and the mode of action of some *α*-KTxs isolated from different potent North African *Androctonus* venoms. In particular, numerous studies on the *Androctonus mauretanicus mauretanicus *venom “manually” extracted, which can be considered as the physiological venom secretion, have greatly contributed to the chemical, immunological, structural, and pharmacological characterization of some highly specific K^+^ channel blockers. With AmmTX3, we have enlarged the *α*-KTx_15_ subfamily and defined more properly its target, the K_v_4 channels. KTX, from the *α*-KTx_3_ subfamily, was finally proven to be a powerful tool used by several international teams to depict the molecular mechanisms of interaction between K^+^ channels and peptide inhibitors, as well as to demonstrate that the binding of K^+^ channel specific scorpion toxins does not take place only on the outer vestibule of the channel pore but also deeper into the selectivity filter. The binding involves a combination of hydrophobic, hydrogen bonding and electrostatic interactions, which induces significant structural rearrangements in both interacting molecules. It was then proposed that structural flexibility of the K^+^ channel and the toxin represent an important determinant of the high specificity of toxin/K^+^ channel interactions [13].
